# Suggestions for how academia can improve the health of medicine**

**DOI:** 10.1590/S1516-31802010000200002

**Published:** 2010-03-04

**Affiliations:** I Physician. Titular professor and head of the Discipline of Emergency Medicine and Evidence-Based Medicine of Universidade Federal de São Paulo — Escola Paulista de Medicina (Unifesp-EPM). Director of the Brazilian Cochrane Center and Director of Associação Paulista de Medicina (APM). E-mail: atallahmbe@uol.com.br

The Brazilian system for postgraduate degrees within the field of medicine is rapidly being transformed into a system for postgraduate degrees in healthcare that congregates all types of professionals. It does not take much effort to see that, even in programs created to develop research in medical specialties, physicians are a minority.

This has come about for various reasons, among which the requirement for exclusive dedication that goes with awards of research grants, which provide a monthly income of around R$ 1,020.00 to R$ 1,530.00. In practice, this situation ends up being of interest for non-physicians at the start of their careers.

In addition, the criteria for judging curriculums in medical schools have been governed by the same criteria as used in basic medical fields regarding publication in journals with a high impact factor, independent of the quality of studies and the time required for developing them. In other words, the journal in which publication is achieved is evaluated and not the article that is published.

It is known that clinical studies generally require several years to reach conclusions and the respective publication. This set of factors forms part of a process that has been progressively weakening academic recognition and the power of dedicated clinicians in medical schools themselves.

From a practical point of view, young physicians have great interest in medical residence, through which they can learn a lot. However, they have little stimulus towards research because they have low chances of success, particularly in competition with non-physicians, given that the criteria used are not the same regarding knowledge, skills and attitudes that are of interest within medicine. This is knowledge that, on average, it takes physicians ten years to acquire, but then they would be starting a career from scratch. During this time, non-physicians will have had the time to achieve master's and doctoral degrees and to (deservedly) enrich their Curriculum Vitae. The consequence of this is that physicians lose strength in academic fields and hence also in professional fields, as well as deviating their career objectives away from clinical activity.

For many years, the Coordination Office for Advancement of University-level Personnel (Coordenação de Aperfeiçoamento de Pessoal de Nível Superior; Capes) has been endeavoring to put the implementation of scientific evidence into practice. It can be said that this is currently one of the greatest challenges within medicine and healthcare. One of its initiatives has been to stimulate the offer of master's programs as professional qualifications.* This has caused reactions, most obviously among individuals with little medical knowledge, who take the view that there is only one form of science, pure science, independent of applications of its results that are more immediate.

Fortunately, they are wrong. Everything requires improvement, particularly when practice shows that new technologies do not correspond to needs. The revolution generated by the evidence-based medicine and healthcare movement is the best proof of such thinking.

More recently, Capes issued an announcement calling for educational institutions with accredited medical residence programs to create master's programs for Professionals Associated with Medical Residence.^1^ Thus, an opportunity has arisen for concomitantly improving medical residence, providing greater density of knowledge, seeking out new knowledge, preparing residents for specific improvements, interesting medical residents in quality clinical research and, through this, advancing the possibility of academic inclusion for physicians within different sectors of society (given that “the system” has excluded them), even within academia itself.

Universidade Federal de São Paulo (Unifesp), along with other major medical schools that we will not cite here because the process is still ongoing in their cases, responded rapidly to the announcement by Capes and forwarded projects for approval. Unifesp has created a Unified Master's Program for Professionals Associated with Medical Residence that concentrates on two major themes: health promotion and healthcare technologies. Thus, from residents’ second year onwards, starting with questions structured during their own residence, they will be able to develop skills in clinical research, critical reading of the literature and development of clinical trial projects, observational studies, technology assessments, clinical guidelines and manuals based on good scientific evidence, within the fields of their personal interests and those in which they will work. In other words, in order to specialize, residents need to acquire the capacity to select and develop new technologies that are based on scientific evidence and are worthwhile for the localities and population where they will work.

The reception for the project was encouraging. Hundred of academic advisors offered to participate in the program. At the same time supervisors of the residence program will be able to improve their abilities as advisors or co-advisors for relevant clinical research projects and thus increase their chances of academic inclusion, which is vital for the future of Brazilian medicine. A bridge between practice and research in the field of medicine has been discerned. This is not expected to affect the practical activities within medical residence, and the quality of scientific products also has to be maintained. Nevertheless, at the same time, it gives the opportunity for physicians to critically deepen their knowledge within the field of their practical interest.

Now it is up to Capes to use its skills in order to administrate this response from academia. For this, a specific assessment committee for the professional master's program needs to be constituted. This would need to be formed by trained professionals with a sympathetic attitude towards the initiative: otherwise, in unsympathetic hands, this important initiative might be placed on the back burner or extinguished.

In turn, academia needs to stop judging scientific production from the field of clinical medicine using the same criteria as used for basic fields. Every researcher knows that weights of very different orders of magnitude cannot be measured using the same type of balance. One simple way to proceed would be to assess the Curriculum Vitae of physicians who are candidates for academia with the same weighting for each of their specific obligations, i.e. to give equal weights to teaching, research and extension (or medical care) activities, taking an arithmetic mean from these results. In other words, greater scientific training should be required from medical residence, while valuing professional competence at the time of the physician's academic assessment: obvious but fundamental for the whole of society.

Send your letters to the editor's office. Thank you.

## Footnotes

* We recommend that the Ministry of Education's Regulatory Ordinance No. 17, of December 28, 2009, should be consulted. Available from: http://semesp.org.br/portal/pdfs/juridico2010/Portarias/POTARIA_17_28_11_09.pdf
